# The Genetic Basis of Cognitive Impairment and Dementia in Parkinson’s Disease

**DOI:** 10.3389/fpsyt.2016.00089

**Published:** 2016-05-20

**Authors:** Lucy M. Collins, Caroline H. Williams-Gray

**Affiliations:** ^1^John Van Geest Centre for Brain Repair, University of Cambridge, Cambridge, UK

**Keywords:** Parkinson’s disease, cognition, dementia, genetics, COMT, MAPT, APOE, GBA

## Abstract

Cognitive dysfunction is a common feature of Parkinson’s disease (PD) with mild cognitive impairment affecting around a quarter of patients in the early stages of their disease, and approximately half developing dementia by 10 years from diagnosis. However, the pattern of cognitive impairments and their speed of evolution vary markedly between individuals. While some of this variability may relate to extrinsic factors and comorbidities, inherited genetic heterogeneity is also known to play an important role. A number of common genetic variants have been identified, which contribute to cognitive function in PD, including variants in catechol-*O*-methyltransferase, microtubule-associated protein tau, and apolipoprotein E. Furthermore, rarer mutations in glucocerebrosidase and α-synuclein and are strongly associated with dementia risk in PD. This review explores the functional impact of these variants on cognition in PD and discusses how such genotype–phenotype associations provide a window into the mechanistic basis of cognitive heterogeneity in this disorder. This has consequent implications for the development of much more targeted therapeutic strategies for cognitive symptoms in PD.

## Introduction

Parkinson’s disease (PD) is typically characterized as a movement disorder with the cardinal motor features of bradykinesia, rigidity, and rest tremor reflecting dysfunction within dopaminergic nigrostriatal circuitry. However, non-motor symptoms constitute a significant part of the symptom burden in PD, reflecting more widespread pathology throughout both the brain and the peripheral and enteric nervous systems. Among the most relevant of these non-motor symptoms in terms of quality of life and planning care requirements is cognitive dysfunction. This evolves through the course of the disease and dementia ensues in nearly 50% of PD patients by 10 years into their illness (1). The factors that underlie this evolution are not fully understood, but pathologically the dementing process seems to involve Lewy body deposition and Alzheimer’s type changes in cortical brain regions (2) as well as widespread cholinergic deficits (3).

**Figure 1:**
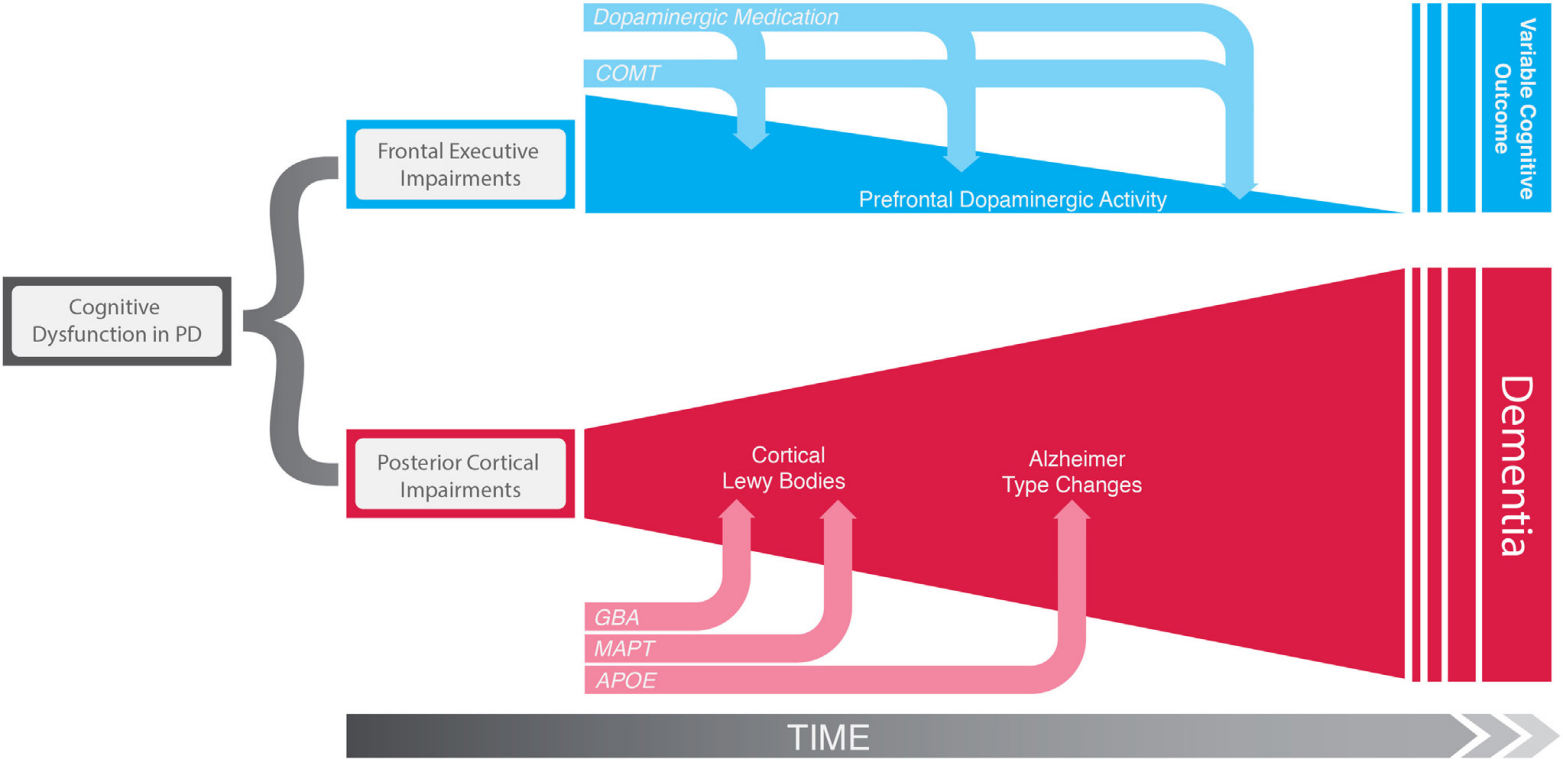
**Schematic representation of the two distinct cognitive syndromes of Parkinson’s disease**. “Frontal executive” impairments in early disease appear to be a consequence of a hyperdopaminergic state in the prefrontal cortex, which is in turn modulated by *COMT* genotype and dopaminergic medication. These deficits can get better or worse over time but are not associated global cognitive decline and dementia risk. In contrast, early deficits on more posterior cortically based cognitive tasks do not have a dopaminergic basis, but reflect the early stages of a dementing process due to Lewy body deposition and Alzheimer’s type changes in posterior cortical areas. This irreversible pathological process is influenced by early on by *GBA* mutations, *MAPT* H1/H2 haplotypes, and at a later disease stage by variation in *APOE*.

Cognitive deficits in PD are heterogeneous in terms of the domains affected including executive dysfunction, memory, and visuospatial impairments. There is also considerable variation in the speed at which cognitive deficits develop, with some patients progressing rapidly to dementia while others have a slower course (1). To some extent, this course is predictable early on in the disease: in an incident population-representative PD cohort followed up for 10 years from diagnosis, patients who were older at diagnosis with more motor impairment and early dysfunction on neuropsychological tests with a posterior cortical basis (semantic fluency and pentagon copying) progressed more rapidly to dementia. In contrast, more frontostriatally based executive deficits were not associated with earlier dementia and, in fact, even improved in some patients as the disease progressed (1) (Figure 1). Similarly, verbal fluency and silhouette perception impairments preceded onset of dementia in an independent longitudinal study (4). Further support for the concept that posterior cortical dysfunction predicts dementia comes from neuroimaging studies with longitudinal follow-up demonstrating that hypometabolism (5) and atrophy (6) in posterior cortical regions precede cognitive decline.

Thus, mild cognitive impairment in PD (PD-MCI) *per se* is not necessarily a prodrome of dementia: some individuals with PD-MCI remain cognitively stable over time and some revert back to normal cognitive function (7, 8). While cognitive phenotype is helpful in predicting prognosis in these “PD-MCI” cases as discussed above, knowledge of genetic variation may also be useful in this regard. The underlying genetic basis of PD is complex: a small proportion of PD cases are accounted for by monogenic forms of PD inherited in a Mendelian fashion (e.g., related to mutations in α-synuclein (*SNCA*), leucine-rich repeat kinase 2 (*LRRK2*), *parkin*, Parkinson’s disease-associated kinase-1 (*PINK-1*), *DJ-1*, *ATP13A2*), but numerous susceptibility genes and loci have also been identified (at least 24) (9), which contribute to the risk of idiopathic PD. In this article, we will review how this complex genetic heterogeneity maps onto phenotypic cognitive heterogeneity of PD. We will first discuss the cognitive characteristics of Mendelian forms of PD. We will then focus on two PD susceptibility genes, namely, glucocerebrosidase (*GBA*) and microtubule-associated protein tau (*MAPT*), both of which influence progression to dementia in PD. Finally, we will discuss candidate genes implicated in modulating cognition in PD but not associated with increased risk of development of PD *per se*. These include catechol-*O*-methyltransferase (*COMT*) and apolipoprotein E (*APOE*). A better understanding of these genetic influences on cognition in PD will lead to improved patient stratification and could guide novel therapeutic strategies for cognitive dysfunction in PD, better targeted at the most relevant underlying pathogenic pathways.

## Insights from Monogenic Parkinson’s Disease

Although most cases of PD are idiopathic, 3–5% of cases are caused by a single gene variant (10). Genes associated with autosomal dominantly inherited PD include *SNCA* (11–13) and *LRRK2* (14, 15), and more recently eukaryotic translation initiation factor 4 gamma-1 (*EIF4G1*) (16) and vacuolar protein sorting-associated protein 35 (*VPS35*) (17) have been identified. Autosomal recessive forms of PD are associated with mutations in *Parkin* (Parkin E3 ubiquitin ligase) (18), *PINK-1* (19), *DJ-1* (*PARK7*, Parkinson protein 7) (20). These different genetic forms of PD vary in their clinical and cognitive phenotypes (see Table 1).

**Table 1:** **Monogenic PD genes**.

	Clinical phenotype	Associated neuropathology	Reference
**AUTOSOMAL DOMINANT**			
*SNCA*	Early onset rapidly progressive parkinsonism with dementia, behavioral impairments, autonomic dysfunction	Widespread Lewy body pathology in brainstem and cortex; neuronal loss in hippocampus	(11, 12, 21–23)
*LRRK2*	Similar to idiopathic PD, but dementia less common	Nigrostriatal degeneration ± cortical involvement; Lewy bodies in most with G2019S mutation, often absent with rarer mutations	(14, 15, 24–28)
*EIF4G1*	Late onset slowly progressive DOPA responsive parkinsonism with preserved cognition	Lewy body pathology in brainstem and cortex	(16)
*VPS35*	Tremor dominant DOPA responsive parkinsonism, variable cognitive, and behavioral features	Pathology uncertain	(17, 29)
**AUTOSOMAL RECESSIVE**			
*Parkin*	Early onset DOPA responsive parkinsonism with dystonia and diurnal fluctuation, slow progression, no/minimal cognitive impairment	Nigrostriatal degeneration, minimal cortical involvement, no Lewy bodies	(18, 30, 31)
*PINK1*	Early onset slowly progressive DOPA responsive parkinsonism ± affective and psychiatric symptoms	Lewy pathology and neuronal loss in the substantia nigra pars compacta, but sparing the locus coeruleus (1 case)	(19, 24, 32, 33)
*DJ1*	Early onset slowly progressive DOPA responsive parkinsonism, no/minimal cognitive impairment	Pathology uncertain	(20)

Some of these genotype–phenotype associations are very well established and provide useful insights into pathogenesis. For example, monogenic forms of PD caused by mutations within, or a change in dose of, the *SNCA* gene implicate α-synuclein in the development of dementia in PD. *SNCA* mutations, including A53T, A30P, and E46K, are often associated with an early onset severe clinical phenotype with cognitive decline and behavioral change as well as autonomic dysfunction (21–23). Similarly, individuals with *SNCA* triplications have early onset rapidly progressive parkinsonism and dementia (11). The pathology in these cases is characterized by widespread Lewy body not only in the brainstem but also in the cortex, as well as neuronal loss in the hippocampal CA2/3 region (11, 24). However, *SNCA* duplication leads to PD with a more benign phenotype, similar to idiopathic PD (34). In contrast, *Parkin*-associated PD is a more “pure” motor disorder with a very low incidence of dementia. A meta-analysis of available phenotypic data in *Parkin* mutation carriers indicated that only 7% of 58 cases carrying 1 mutant allele developed dementia over 12 years of follow-up, with incidence rates being even lower in those carrying 2 mutant alleles at 1% of 232 cases over 19 years (30). This is consistent with neuropathological findings in *Parkin*-associated PD, which indicate that the disease is largely restricted to the nigrostriatal tract with the absence of Lewy bodies (31). *LRRK2* mutations represent the commonest cause of autosomal dominantly inherited PD (25), and hence the clinical phenotype has been well described, particularly in association with the most frequently occurring *G2019S* mutation. The clinical phenotype is similar to idiopathic PD (26), although the prevalence of dementia is reported to be lower (27,  28). Most *LRRK2 G2019S* PD cases have the typical pattern of brainstem pathology with Lewy bodies with variable cortical involvement, but Lewy bodies are not a universal finding, and are present in less than half of those with *LRRK2* mutations other than G2019S (24), in keeping with the more benign phenotype when compared to *SNCA*-associated PD. Other observed but less well-replicated genotype–phenotype associations include a high prevalence of psychiatric symptoms in *PINK-1* families (32) and learning difficulties and cognitive impairment in a Swiss family with the D620N mutation in *VPS35* causing early onset tremor dominant PD (29).

Although these observations are of interest, in general there is a paucity of longitudinal data on phenotypic characteristics in Mendelian forms of PD. A meta-analysis in 2010 identified 119 studies reporting information on non-motor symptoms in 990 cases with monogenic forms of PD, but the available phenotypic data were limited, with dementia frequency being reported in only 54% of studies, and most studies did not clearly distinguish between point and lifetime prevalence of non-motor symptoms, thus making interpretation of their data difficult. With these important caveats, the meta-analysis confirmed the highest prevalence of dementia among *SNCA* mutation carriers (approximately 25%) and the lowest among *Parkin* mutation carriers (approximately 3%) (35).

Hence, although these monogenic forms provide some insight into the pathogenesis of PD dementia, a more systematic evaluation of their phenotypic characteristics is needed to clarify how they contribute to the cognitive heterogeneity of PD.

### Glucocerebrosidase

Mutations in the glucocerebrosidase gene, *GBA*, cause the autosomal recessive storage disorder Gaucher’s disease, a lysosomal storage disorder, but the observation that parkinsonism is frequently observed in this disease and in family members of Gaucher’s patients (36), in association with Lewy body deposition in the brainstem and cortex, led to the recognition of an increased frequency of heterozygote GBA mutations in PD cases versus controls. These mutations occur even in those with seemingly sporadic disease and have now been established as the commonest genetic risk factor for PD identified to date (37). GBA mutations are estimated to account for around 3–4% of PD cases (38, 39) with much higher frequencies of around 15% in Ashkenazi Jewish populations (38). However, these mutations also occur in around 3% Ashkenazi Jewish individuals and 1% of non-Jewish individuals (38, 39), hence their classification as a susceptibility factor for PD rather than a monogenic cause of the disease.

There is now clear evidence that *GBA*-associated PD has a more aggressive phenotype than idiopathic PD, particularly in terms of progression to dementia. In cross-sectional studies, the prevalence of dementia in *GBA*-PD cases is reported to be around 50% (39, 40), compared to a dementia prevalence of 24–31% in idiopathic PD cases (41), and this difference is particularly striking given that many *GBA*-associated cases are young onset (39). GBA mutations have also been reported to be associated with a younger onset of Dementia with Lewy Bodies, as well as male gender (42), although this gender association has not been replicated and, in fact, an opposite gender effect has been reported for PD with *GBA* mutations being more common in women (40). Longitudinal studies have confirmed a faster progression to dementia in PD cases carrying *GBA* mutations (43, 44), with an odds ratio for dementia of 4.6 (95% CI 1.3–15.9) for *GBA*-PD compared to idiopathic PD in a population-representative cohort followed up for 10 years from diagnosis (43). Cognitive deficits are also described in patients with Gaucher’s disease and healthy carriers of *GBA* mutations (45, 46) and may be an early indicator of neurodegenerative pathology in these cases.

The mechanism underlying this association between *GBA* mutations and PD/PD dementia is still not entirely clear. *GBA* is a lysosomal enzyme responsible for the breakdown of glucosylceramide, resulting in the accumulation of glucosylceramide in cells (47). Pathologically, PD-*GBA* cases are characterized by limbic or neocortical Lewy body deposition (48), and colocalization of glucocerebrosidase with Lewy bodies in these *GBA* mutation carriers has been demonstrated (49), supporting the theory that mutant glucocerebrosidase may contribute directly to increased α-synuclein aggregation (50, 51). It has been proposed that accumulation of the substrate glucocerebroside in the lysosome, due to impaired function of the enzyme, acts as a backbone for α-synuclein aggregation. This in turn leads to compromise of *GBA* activity and lysosomal function and a consequent increase in α-synuclein aggregation in a positive feedback loop (51). Further understanding of these mechanisms may lead to the development of novel disease-modifying therapies targeted at *GBA* to slow progression to dementia in PD. However, the overall impact of *GBA* mutations on the incidence of dementia in PD remains low given that these mutations account for only 3–4% of PD cases (38, 39), and it is unclear how relevant such therapeutic strategies might be in idiopathic PD.

### Microtubule-Associated Protein Tau

Microtubule-associated protein tau is a protein involved in microtubule assembly and stabilization, which forms pathological aggregates in a number of neurodegenerative diseases. The gene is encoded on chromosome 17q21 in the center of a 900-kb long fragment, which is inverted in a proportion of the population due to the divergence of two chromosomal lineages (H1 and H2) more than 3 million years ago with no recombination since (52). The inverted H2 haplotype accounts for approximately 25% of haplotypes in those with European ancestry. *MAPT* H1/H1 genotype has been firmly established not only as a risk factor for tauopathies, including progressive supranuclear palsy (PSP) and corticobasal degeneration (CBD), but also associated with increased PD risk (53). Although significant tau pathology is not found in the majority of idiopathic PD cases at postmortem, there is clear evidence that tau interacts with α-synuclein in Lewy body formation. Tau epitopes are found within Lewy bodies on immunostaining of PD postmortem brains (54), α-synuclein fibrils have been shown to induce polymerization of tau into hyperphosphorylated aggregates *in vitro* (55), and transgenic mice overexpressing α-synuclein have increased levels of hyperphosphorylated tau colocalizing with α-synuclein deposits (56). Thus, *MAPT* variation is a good candidate to influence the development of dementia in PD given the widespread cortical Lewy body deposition which characterizes this state.

Association between the *MAPT* H1/H1 genotype and cognitive dysfunction in PD was first reported in 2007 in a longitudinal study of a population-representative incident PD cohort (*n* = 108): over a mean (SD) follow-up of 3.5 (0.7) years from diagnosis, age was strongly correlated with cognitive decline in H1 homozygotes but cognitive performance remained unchanged in H2 carriers, regardless of advancing age. In the same CamPaIGN cohort at 5 years from diagnosis, all but one of the PD cases who had developed dementia carried the H1/H1 genotype (18/65 of H1/H1 versus 1/34 of H2 carriers), with an estimated odds ratio (with adjustment for age and other confounding factors) for dementia of 12.1 (57), strikingly greater than the estimated odds ratio for PD susceptibility for *MAPT* of 1.4 (53), suggesting that *MAPT* H1 has a much more profound influence on cognitive function in early PD than on risk of disease *per se*. The 10-year follow-up data from CamPaIGN continued to support an association between H1/H1 genotype and progression to dementia, although the magnitude of the effect seems to lessen with increasing disease duration (age-adjusted hazard ratio 2.9) (1). While this association has been replicated by other groups, including a large case–control study reporting a much stronger association with H1 haplotype for PD dementia (OR = 3.73; *P* = 0.002) than for PD without dementia (OR = 1.89; *P* = 0.04) (58), some studies have failed to find such an association (59, 60). The discrepancy may reflect the nature of the cohorts assessed, with negative associations in prevalent cohorts with longer disease durations, suggesting that the predominant effect of the *MAPT* H1 variant may be to accelerate cognitive decline within the early years of the disease.

Further support for this concept comes from functional MRI studies in patients with early PD, demonstrating association between *MAPT* H1/H1 and reduced brain activation in medial temporal and parietal regions during memory and visuospatial tasks, respectively, but no association with frontostriatal function (61, 62). Hence, the *MAPT* H1/H1 effect seems to be specific to posterior cortical circuitry, and given the observations that posterior cortical deficits predict early dementia in PD (discussed above), this genetic variant may be a critical driver of this dementing pathway (see Figure 1).

Evidence from postmortem studies supports the hypothesis that the *MAPT* H1 variant exerts it phenotypic effects through promoting protein aggregation and Lewy body formation. In a cohort of 22 dementia with Lewy body cases, total Lewy body counts were found to be significantly higher in H1/H1 (*n* = 12) versus H2 (*n* = 10) carriers matched for demographics and clinical variables, with no between-group difference in Alzheimer’s type pathology (63). Furthermore, in a large postmortem series of cases with Alzheimer’s disease (AD), Lewy body disease or vascular pathology (*n* = 762), the *MAPT* H1 variant was associated with higher cortical Lewy body counts and reduced Alzheimer’s type changes (64). The functional mechanism by which the *MAPT* H1 variant might lead to changes in protein aggregation is unknown, but increased expression levels of total tau or of four repeat tau isoforms has been reported in PD as well as the tauopathies (65–67), and the H1 haplotype is associated with an increase in four repeat tau transcription in PD brain (57, 68).

### Catechol-*O*-Methyltransferase

The COMT enzyme is a key regulator of synaptic dopamine levels, particularly in the frontal cortex where the dopamine transporter (DAT) is rarely expressed (69). The *COMT* gene contains a common functional polymorphism of valine (val) for methionine (met) at codon 158, which alters the thermostability of the enzyme, resulting in a reduction in activity of 40% in human frontal cortex in met versus val homozygotes (70). Healthy subjects with low activity *COMT* genotypes (met/met, with putatively higher prefrontal dopamine levels) show improved performance on working memory (WM) and attentional control tasks activating the prefrontal cortex (71–73). Hence, *COMT* val^158^met is a good candidate for modulating the frequently observed executive deficits in PD, which are known to be mediated by dopaminergic dysfunction in frontostriatal networks (74).

This genetic variant has indeed been shown to alter performance on such tasks in PD, although the direction of the effect is opposite to that seen in controls. Specifically, in a cohort of patients with early PD (*n* = 288), an increase in met alleles (i.e., lower COMT activity and putatively higher dopamine levels) was associated with impaired performance on the CANTAB one-touch Tower of London, a planning task dependent on prefrontal cortical areas, and the impairment was greatest in patients on dopaminergic medications (75). This association between *COMT* val^158^met and executive function has since been replicated in an independent early PD cohort (CamPaIGN study), although the effect size was small (57). A further study found evidence of an interactive effect of *COMT* met alleles and dopaminergic medication on executive performance, although it did not demonstrate a main effect of *COMT* possibly due to insufficient power (76). Functional MRI studies further support a role for *COMT* val^158^met in mediating executive function in PD, demonstrating that impaired performance on both planning and attentional control tasks in met homozygotes is associated with reduced BOLD activation in frontoparietal networks (77, 78), and revealing an interaction between genotype and dopaminergic medication (62), which has potential implications for planning treatment in PD patients with significant executive dysfunction.

**Figure 2:**
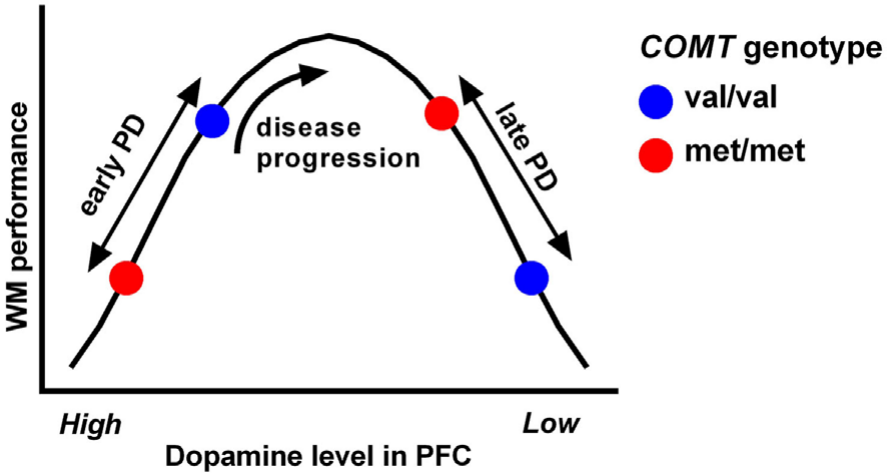
**The relationship between working memory (WM) performance and dopamine levels in the prefrontal cortex follows an inverted *U*-shaped curve**. Behavioral and functional imaging data from PD patients indicates that an individual’s position on the curve is dependent on their stage of disease as well as their *COMT* val^158^met genotype (which determines the activity of the COMT enzyme). Hence, the relationship between executive function and COMT genotype in PD is complex, and executive deficits may improve rather than worsen in certain genotypic groups as the disease progresses. Reproduced from Williams-Gray et al. (84).

The detrimental effect of *COMT* met alleles on executive performance in early PD (in contrast to the positive effect in healthy controls) seems likely to relate to an “overload” of dopamine in the prefrontal cortex, which can be further exacerbated by dopaminergic medication. This hypothesis is consistent with [18F]DOPA-PET studies indicating a hyperdopaminergic state in prefrontal areas in early PD (79, 80) (presumably compensating for nigrostriatal dopamine loss), as well as a more recent [18F]-DOPA study indicating that *COMT* met alleles are associated with reduced dopamine metabolism and higher synaptic dopamine levels in frontal brain regions in early PD patients (81). Indeed, it is well established in the animal literature that there is an inverted *U*-shaped relationship between executive function and dopamine levels in the frontal cortex, with high and low synaptic dopamine levels causing impaired performance (82). As PD progresses, dopamine levels in the prefrontal cortex decline (83) thus resulting in a shift in position on the inverted *U*-shaped curve, and an alteration in the relationship between *COMT* genotype and executive performance with time (Figure 2), which has been demonstrated when comparing subgroups of patients with early versus late PD (57). Furthermore, longitudinal analysis in the CamPaIGN incident PD cohort has shown that executive performance improves in met/met individuals over 5 years of disease progression, in contrast to other *COMT* genotypic groups. So, this genetic variant, while modulating executive heterogeneity in PD through its effects on frontal dopaminergic networks, is not necessarily associated with poor cognitive outcomes in PD and, in particular, does not appear to be associated with dementia risk (57).

### Apolipoprotein E

Association between the *APOE* gene and susceptibility to AD is well established (85). There is an overlap between AD and PD both clinically and pathologically: dementia and extrapyramidal features occur in both, and neuronal loss and aberrant protein aggregation are common pathological features; and hence, a possible association of *APOE* with susceptibility to PD and PD dementia has been debated for some time. *APOE* has three major alleles: *APOE-*ɛ*2, APOE-*ɛ*3*, and *APOE-*ɛ*4*. *APOE-*ɛ*4* is associated with both AD risk and lower age of onset, whereas *APOE-*ɛ*2* is protective (85). The functional basis of these effects is thought to be mediated at least in part through altered amyloid metabolism, but *APOE* has also been implicated in the molecular pathway of α-synuclein-mediated neurodegeneration (86).

A large meta-analysis of over 4000 cases and 10,000 controls has suggested an over-representation of *APOE-*ɛ*2* carriers among PD patients compared to controls, although the odds ratio was very modest at 1.16 (95% CI 1.03–1.31) (84), and association between *APOE* and PD risk has not been replicated in further large scale genetic association studies (87) or genome-wide association studies in PD. Further meta-analysis indicated an over-representation of *APOE-*ɛ*4* carriers among PD dementia cases (*n* = 501) compared to PD non-dementia cases (*n* = 1145) [OR 1.74 (1.36–2.23)] (84). However, the validity of this finding is limited by the small number of PDD cases in each study, the significant heterogeneity of odds ratios between studies, and evidence of publication bias (84). Perhaps most importantly, such cross-sectional studies are probably misleading because any effect of *APOE-*ɛ*4* is likely to be time-dependent analogous to its effect in AD. Longitudinal analyses should be more informative, but only a small number of such studies have been published to date, and again findings have been inconsistent.

The effect of *APOE-*ɛ*4* carrier status on longitudinal cognitive decline in PD was investigated in the CamPaIGN cohort (*n* = 107) over a 5-year period from diagnosis, and no evidence for association with rate of change in MMSE scores, age-dependent cognitive decline, or incidence of dementia was found (84). Similarly, no associations between *APOE* genotype and development of dementia were found in another incident PD cohort (*n* = 64) followed up for a mean of 9.7 years from diagnosis (88). In contrast, a study of 212 PD patients found more rapid cognitive decline (Mattis Dementia Rating Scale) over 4 years in *APOE-*ɛ*4* carriers compared to non-carriers, but these were more advanced cases at study entry with a mean disease duration of 7 years (60). In the same study, *MAPT* H1/H1 genotype was not associated with cognitive decline over time, although there was an association with memory performance (60). Hence, the disparity between studies may reflect the fact that these genetic variants have time-dependent effects on cognition which vary with disease stage: *MAPT* seems to have its greatest impact on cognitive decline in early PD, whereas *APOE* may have a more pronounced effect in later disease. This raises the question of whether association between *APOE* genotype and dementia in PD reflects the development of Alzheimer’s type pathology in the aging brain. Indeed, a recent study of 232 PD cases reporting association between *APOE-*ɛ*4* and dementia status (OR 5.15) found Alzheimer’s type pathology including moderate to high frequency neuritic plaques and Braak tau stage ≥3 in 90% of *APOE-*ɛ*4* carriers coming to postmortem (*n* = 10) compared to 43–53% of non-carriers (*n* = 19) (89). However, postmortem examination over 900 dementia cases and controls genotyped for *APOE* has demonstrated that *APOE-*ɛ*4* frequency is increased not only in cases with pathologically confirmed AD (38.1%) and mixed Lewy body-AD pathology (40.6%) but also in those with “pure” DLB (31.9%) and “pure” PDD (19.1%) compared to controls (7.2%), indicating that the contribution of *APOE-*ɛ*4* to the dementing process in PD is not mediated entirely through effects on amyloid metabolism and Alzheimer’s type changes (90).

## Discussion and Conclusion

As our knowledge of the genetic basis of PD continues to expand, it is becoming increasingly apparent that genetic factors are important not only in determining an individual’s susceptibility to the disease but also how their disease evolves phenotypically (Table 2). Clinical evaluation of cases with Mendelian forms of PD has been informative to some extent, in particular, in terms of corroborating the importance of α-synuclein in the pathogenesis of dementia in PD, but longitudinal data still remains limited in many monogenic forms of PD, and more detailed follow-up data from these rare cases, ideally with postmortem evaluation, would be very valuable. GBA mutations are the most common genetic risk factor for PD, and consequently phenotypic data on *GBA*-PD cases are rapidly accumulating, suggesting that these cases have an aggressive form of the disease with early onset dementia and florid cortical Lewy body pathology. This knowledge has prompted a wealth of research into the mechanistic relationship between glucocerebrosidase, lysosomal function, and α-synuclein aggregation, which may lead to novel therapeutic strategies.

**Table 2:** **Genes contributing to cognitive heterogeneity in PD**.

Genetic variant	Association with PD risk	Phenotypic effect	Proposed mechanism	Reference
*GBA* mutations	Yes	More rapid progression to dementia	Impaired GBA activity, accumulation of glucocerebroside and lysosomal dysfunction, consequent increase in α-synuclein aggregation	(39, 40, 43, 44, 48)
*MAPT* H1/H2 hapotype	Yes	Faster global cognitive decline and higher dementia risk in H1 carriers, particularly in early PD	Increased expression of four repeat tau, increased protein aggregation, and Lewy body formation	(1, 57, 58, 65–67)
*COMT* val^158^met polymorphism	No	Met alleles associated with impaired executive function in early PD, though may improve with disease progression	Increased prefrontal dopamine levels in met carriers leading to “overload” effect	(57, 91)
APOE ɛ4 allele	No	Higher dementia risk, particularly in later stage PD	Altered amyloid metabolism promoting Alzheimer’s type pathology but also implicated in “pure” Lewy body disease	(60, 84, 88–90)

The approach of investigating candidate genes within putatively relevant neurobiological pathways mediating cognitive dysfunction in PD is of course inherently biased, but nonetheless has yielded some very interesting insights into how cognitive deficits evolve in this disorder, and in particular has helped to define distinct cognitive syndromes (57). The frontal dysexecutive syndrome is related to dopaminergic dysfunction in frontostriatal networks and modulated by *COMT* genotype and does not necessarily indicate the onset of a dementing process. In contrast the posterior cortical syndrome is an age-dependent process modulated by *MAPT* and *APOE* genotypes, which is likely to reflect irreversible protein aggregation and neuronal dysfunction in the temporal and parietal cortices, and evolves into clinical dementia (Figure 1). Further support for this idea of distinct cognitive syndromes in PD has recently come from a study which used functional MRI to further interrogate the association between *COMT*, *MAPT*, and *APOE* genotypes and brain function in the ICICLE-PD cohort of newly diagnosed PD patients (*n* = 168). This study used tasks designed to probe executive, visuospatial, and memory domains and demonstrated that these three genes had dissociable effects on performance in the different cognitive domains associated with regionally specific changes in cortical activation. Specifically, *COMT* val^158^met was found to interact with levodopa dose to modulate activation in frontostriatal regions during the executive task, *MAPT* H1 versus H2 haplotype-modulated activation in parietal regions during the visuospatial task, and *APOE* genotype-modulated activation in temporoparietal regions during the memory encoding task (62). This concept of different cognitive syndromes in PD has practical implications in term of planning therapy in the clinic, for example, those with a predominantly dysexecutive syndrome in early PD may benefit from a reduction in dopaminergic therapy, whereas those with early posterior cortical deficits warrant close follow-up for an emerging dementia, with a low threshold for starting anticholinesterase inhibitors.

Genetic factors differ not only with respect to the cognitive domains that they influence but also in terms of their temporal relationship with cognition. The relationship between *COMT* val^158^met and executive function in PD can vary over time, both according to disease stage (and underlying frontal dopamine levels) and secondary to changes in dopaminergic medication (57). The dementing process appears to be accelerated in early disease by *MAPT* H1/H1 genotype, although ultimately the same process occurs at a delayed rate in H2 carriers (1); hence, studies in later disease do not necessarily detect a phenotypic effect. In contrast, there is evidence to suggest that *APOE* genotype is associated with the development of dementia in later disease (60), but an impact on early dementia in PD has not been demonstrated in longitudinal studies (84, 88). This highlights the importance of longitudinal rather than cross-sectional studies to gain a true picture of the relationship between genetic factors and phenotypic variation in PD.

The advent of new technologies allowing whole-genome investigations, such as genome-wide association studies (GWAS) and whole-exome sequencing of large cohorts, has led to the recent discovery of a number of new unanticipated genetic loci associated with PD. These include signal-induced proliferation-associated 1 like 2 (*SIPA1L2*), inositol polyphosphate-5-phosphatase F (*INPP5F*), microRNA 4697 (*MIR4697*), GTP cyclohydrolase (*GCH1*), vacuolar protein sorting 13 homolog C (*VPS13C*), and DDRGK domain containing 1 (*DDRGK1*) (9). However, the contribution of genetic variation at these loci to the clinical and cognitive heterogeneity of PD is yet to be explored. Given that the contribution of individual genetic variants may be small, the optimal strategy for the future will be to pool genetic datasets with longitudinal clinical data from multiple centers to provide sufficient power to adequately interrogate genotypic–phenotypic associations. Ultimately, detailed genetic profiling may provide us with a reliable tool for stratification of cognitive prognosis in PD, which will be invaluable for the selection of the most appropriate patients for future trials of therapies to prevent dementia in PD, as well as to better guide management strategies for this devastating aspect of the disease. Furthermore, as our understanding of the genetic basis of cognitive dysfunction in PD evolves, so will our knowledge of the underlying pathophysiological pathways involved, with consequent implications for the development of novel therapies.

## Author Contributions

Both LC and CW-G were responsible for drafting the article, critical revision of the article, and final approval of the version to be published.

## Conflict of Interest Statement

The authors declare that the research was conducted in the absence of any commercial or financial relationships that could be construed as a potential conflict of interest.
